# Functioning and Disability Analysis of Patients with Traumatic Brain Injury and Spinal Cord Injury by Using the World Health Organization Disability Assessment Schedule 2.0

**DOI:** 10.3390/ijerph120404116

**Published:** 2015-04-14

**Authors:** Chia-Ying Kuo, Tsan-Hon Liou, Kwang-Hwa Chang, Wen-Chou Chi, Reuben Escorpizo, Chia-Feng Yen, Hua-Fang Liao, Hung-Yi Chiou, Wen-Ta Chiu, Jo-Ting Tsai

**Affiliations:** 1 School of Public Health, College of Public Health and Nutrition, Taipei Medical University, Taipei 11031, Taiwan; E-Mails: kuo@s.tmu.edu.tw (C.-Y.K.); hychiou@tmu.edu.tw (H.-Y.C.); 2 Office of Medical Affairs, Shuang Ho Hospital, Taipei Medical University, New Taipei City 23561, Taiwan; 3 Graduate Institute of Injury Prevention and Control, College of Public Health and Nutrition, Taipei Medical University, Taipei 11031, Taiwan; E-Mails: peter_liou@s.tmu.edu.tw (T.-H.L.); chang2773@gmail.com (K.-H.C.); 4 Department of Physical Medicine and Rehabilitation, Shuang Ho Hospital, Taipei Medical University, New Taipei City 23561, Taiwan; 5 Department of Physical Medicine and Rehabilitation, School of Medicine, College of Medicine, Taipei Medical University, Taipei 11031, Taiwan; 6 Department of Physical Medicine and Rehabilitation, Wan Fang Hospital, Taipei Medical University, Taipei 11696, Taiwan; 7 School of Occupational Therapy, Chung Shan Medical University, Taichung City 40201 Taiwan; E-Mail: y6312002@gmail.com; 8 Department of Rehabilitation and Movement Science, College of Nursing and Health Sciences, University of Vermont, Burlington, VT 05401, USA; E-Mail: escorpizo.reuben@gmail.com; 9 Swiss Paraplegic Research, Nottwil 6207, Switzerland; 10 Department of Public Health, Tzu Chi University, Hualien City 97004, Taiwan; E-Mail: mapleyeng@gmail.com; 11 Chinese Association of Early Intervention Profession for Children with Developmental Delays, Hualien City 97064, Taiwan; E-Mail: hfliao@ntu.edu.tw; 12 School and Graduate Institute of Physical Therapy, College of Medicine, National Taiwan University, Taipei 10055, Taiwan; 13 Radiation Oncology Department, Shuang Ho Hospital, Taipei Medical University, New Taipei City 23561,Taiwan; E-Mail: 10576@s.tmu.edu.tw

**Keywords:** disability, traumatic brain injury (TBI), spinal cord injury (SCI), World Health Organization Disability Assessment Schedule (WHODAS 2.0), International Classification of Functioning, Disability, and Health (ICF)

## Abstract

The purpose of this study is to compare traumatic brain injuries (TBI) and spinal cord injuries (SCI) patients’ function and disability by using the World Health Organization Disability Assessment Schedule 2.0 (WHODAS 2.0); and to clarify the factors that contribute to disability. We analyzed data available between September 2012 and August 2013 from Taiwan’s national disability registry which is based on the International Classification of Functioning, Disability, and Health (ICF) framework. Of the 2664 cases selected for the study, 1316 pertained to TBI and 1348 to SCI. A larger percentage of patients with TBI compared with those with SCI exhibited poor cognition, self-care, relationships, life activities, and participation in society (all *p* < 0.001). Age, sex, injury type, socioeconomic status, place of residence, and severity of impairment were determined as factors that independently contribute to disability (all *p* < 0.05). The WHODAS 2.0 is a generic assessment instrument which is appropriate for assessing the complex and multifaceted disability associated with TBI and SCI. Further studies are needed to validate the WHODAS 2.0 for TBI and SCI from a multidisciplinary perspective.

## 1. Introduction

Injury is a major global health concern. Violence and injury are the leading causes of death in the Western Pacific region among people aged 5–49 years; most of these fatalities arise from road accidents, falls, drowning, burns, and interpersonal violence [1]. In 2008, the rate of mortality caused by injury in Taiwan was 42.2/100,000 [2], higher than those of Singapore (23.9), Spain (32.5), England (34.8), and Germany (38.8) for the same year [1].

Of the various accidental injuries, central nervous system injuries include traumatic brain injury (TBI) and spinal cord injury (SCI) constitute a major cause of morbidity and mortality. TBI and SCI are important because of their severity and long-term effects. Patients with TBI or SCI can have disabilities and pain for years [3,4]. TBI is a major public health concern worldwide; it is a major cause of death and disability and impairs health-related quality of life [5]. Roughly half of injury-induced deaths arise from TBI [6]. Morbidity associated with severe TBI may result in long-term disability and may burden families and society. According to a systematic review, TBI patients that had a longer inpatient hospital had high injury severity, low physical functioning and a reduced chance of returning to work [7].

SCI, the other common type of injury, is also a severely disabling condition and leads to a range of impairments and secondary health conditions [8]. Patients with SCI experience difficulty participation in society in contexts such as work and leisure activities [9]. Depending on the level of the injury, SCI may cause permanent paralysis and, consequently, other somatic health problems [10]. Therefore, participation in society is a crucial outcome of SCI rehabilitation [11].

Most earlier studies have focused on the medical problems and treatment for TBI and SCI; measures of participation in patients with TBI and SCI remain underdeveloped [12,13]. The World Health Organization Disability Assessment Schedule 2.0 (WHODAS 2.0), published by the World Health Organization (WHO) in 2010, is a generic assessment tool for health and disability and for producing standardized disability levels [14]. The WHODAS 2.0 serves as a basis for comparing disability data among countries. It treats all disorders at par in assessing the levels of functioning and exhibits strong validity, reliability, and cross-cultural applicability in over 30 languages [15]. TBI and SCI are the most severe types of injuries, however there is little evidence to address the difference between these two types of patients in terms of functional, and community outcomes. Therefore, the purpose of this study is to compare these two injury groups of functioning and disability by using the WHODAS 2.0; and to clarify the factors that contribute to disability.

## 2. Methods

### 2.1. Sample

Preliminary data were obtained from a registry of disability evaluation, functional assessment, and provision of social welfare services, which is a database established by the Ministry of Health and Welfare in Taiwan and is based on the International Classification of Functioning, Disability, and Health (ICF) framework [16]. This study was approved by the Joint Institutional Review Board at Taipei Medical University (Approval No. 201004001 and 201205042).

**Figure 1:**
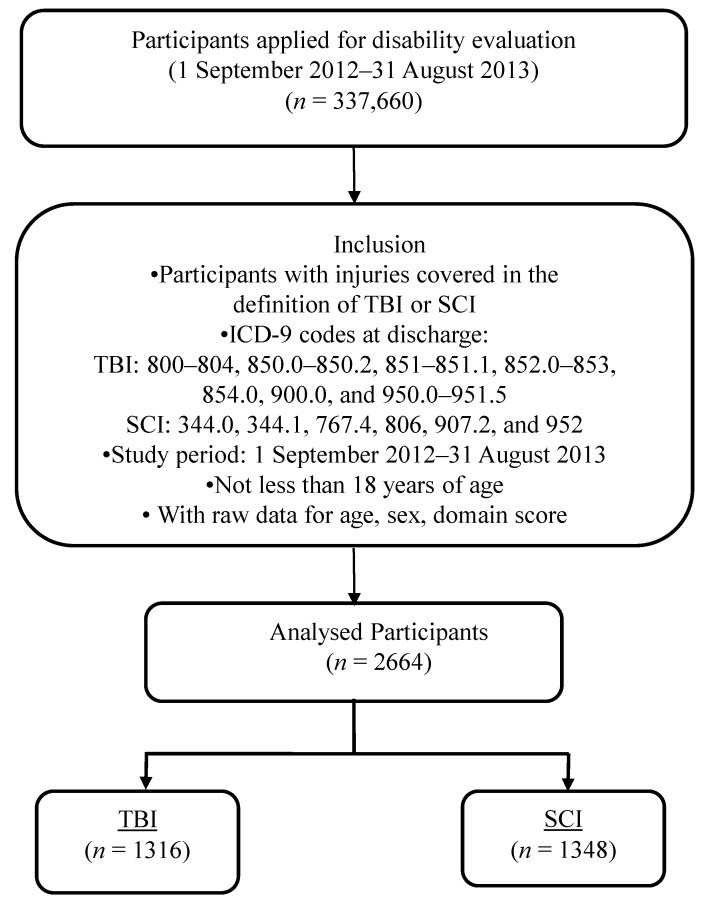
Sample selection flow chart.

Registry applications between September 2012 and August 2013 were collected. Applicants who completed the evaluation procedure and were eventually provided disability benefits by the government of Taiwan were included. Applicants under the age of 18 years, those whose personal or ICF coding data were incomplete, those ineligible for disability determination, and those with both TBI and SCI during the disability evaluation were excluded. TBI cases were identified according to the International Classification of Diseases, Ninth Revision, Clinical Modification codes 800–804, 850.0–850.2, 851–851.1, 852.0–853, 854.0, 900.0, and 950.0–951.5, and SCI cases according to codes 344.0, 344.1, 767.4, 806, 907.2, and 952. This yielded 2664 cases (1793 males and 871 females) of TBI and SCI (Figure 1).

### 2.2. Instruments: Domain and Summary Scores of the WHODAS 2.0

We administered the 36-item questionnaire of the WHODAS 2.0 by interviewing the participants (or their proxies in cases where the patient could not respond). Respondents rated the extent to which their disabilities interfered with their lives in the preceding 30 days on a 5-point scale ranging from 1 (none) to 5 (extreme/cannot do). The domain scores and summary score (the general disability latent variable) were calculated using the 36-item version of the WHODAS 2.0 for student or employed participants, and the 32-item version, which lacks the four items pertaining to work ability, was used for unemployed participants; the scoring algorithm employed is available from WHO. Scores range from 0 (least difficult) to 100 (most difficult), with higher scores indicating a higher level of disability. The algorithm allows for up to 30% of the items to be missing per domain, and substitution of the mean (by domain) was used for imputing missing data. The WHODAS 2.0 consists of six domains, namely domain 1, cognition; domain 2, mobility; domain 3, self -care; domain 4, relationships; domain 5, life activities; and domain 6, participation in society.

Previous studies on discriminant validity of the WHODAS 2.0 in adults with disabilities [17] and SCI cases [13] showed that Cronbach’s α coefficients ranged from 0.61 to 0.97.

### 2.3. Procedure

The diagnosis was made by one physician with a specialty, including a neurosurgeon, physiatrist, or neurologist. The WHODAS 2.0 was evaluated by multiple testers in different hospitals. We trained 7125 professionals, such as physical therapists, occupational therapists, and social workers, during September 2011 and August 2013 for the purpose of disability evaluation. They completed the on-the-job training and were qualified to conduct the evaluations. The internal consistencies (Cronbach’s α) of the domains ranged from 0.90 to 0.93. The collected data were categorized into three sections: (1) In the first section, we recorded sociodemographic variables such as age, sex, place of residence, level of education, ethnicity, socioeconomic status, work status, and family structure; (2) In the second section, which included medical reports, we recorded disability-related variables such as onset of injury, type of injury, diagnosis, and corresponding body functions (b codes) and structural components (s codes) from the ICF. The severity of impairment was determined on the basis of the highest qualifier of the b and s codes (1 = mild, 5%–24% impairment; 2 = moderate, 25%–49% impairment; 3 = severe, 50%–95% impairment; 4 = complete; 96%–100% impairment). For example, if a patient with TBI received an ICF coding of b110.1, b167.2, and b730.3, the severity of impairment is 3, the highest qualifier, which indicates a severe case; (3) In the third section, we recorded the WHODAS 2.0 data (36 items, Traditional Chinese version) [18,19,20]; the scores were determined by trained testers to maximize reliability and consistency.

Statistical analyses were performed using SAS, Version 9.2 (SAS Institute Inc., Cary, NC, USA). Sociodemographic data such as age, education status (no formal education, primary school and above), socioeconomic status (average, middle low and low), place of residence (community dwelling, institutional dwelling), urbanization level (urban, suburban, rural), and severity of dementia-related impairment (mild, moderate, severe, extreme) were represented in numbers and percentages (Table 1). Chi-square analysis was used for comparing the categorical variables of injury-related disability between TBI and SCI. To evaluate the relationship between impairment and WHODAS 2.0 scores, a Poisson regression model was constructed to assess the odds ratio between WHODAS 2.0 scores and sociodemographic variables such as sex, age, and educational level. We considered *p* < 0.05 to be statistically significant.

**Table 1:** Sociodemographic characteristics of participants with traumatic brain injuries (TBI) and spinal cord injuries (SCI).

Variables	TBI (*n* = 1316)		SCI (*n* = 1348)		*p*-value
	*No.*	%	*No.*	%	
Sex					0.293
Male	873	66.34	920	68.25	
Female	443	33.66	428	31.75	
Age (years)					0.446
Mean, SD	58.87	19.139	58.34	16.951	
Median	59.49		58.60		
Education					0.073
No formal education	126	18.39	95	14.18	
Primary	211	30.80	202	30.15	
Above Primary	348	50.80	373	55.67	
Missing	631		678		
Socioeconomic Status					0.111
Average	1254	95.29	1301	96.51	
Middle low & low	62	4.71	47	3.49	
Place of Residence					<0.001
Community dwelling	865	66.13	1000	74.63	
Institutional dwelling	443	33.87	340	25.37	
Missing	8		8		
Urbanization level					0.289
Rural	339	25.76	327	24.26	
Suburban	502	38.15	495	36.72	
Urban	475	36.09	526	39.02	
Severity of impairment					<0.001
Mild	259	19.68	326	24.18	
Moderate	351	26.67	371	27.52	
Severe	235	17.86	506	37.54	
Extreme	471	35.79	145	10.76	

TBI: traumatic brain injury, SCI: spinal cord injury.

## 3. Results

### 3.1. Sample Size

Data on 1316 participants with TBI (873 males and 443 females) and a mean age of 58.87 (SD = 19.14) years and 1348 participants with SCI (920 males and 428 females) and a mean age of 58.34 (SD = 16.95) years were retrospectively reviewed. No significant differences in sex, age, education, and urbanization level were observed between the participants with TBI and those with SCI (Table 1). More participants with SCI resided in community dwellings (74.63%) than those with TBI (66.13%). A significant difference between the participants with TBI and those with SCI was noted in the distribution of severity of impairment (*p* < 0.001): more participants with TBI had extreme severity than those with SCI.

### 3.2. WHODAS 2.0 Scores and Injury Types

Table 2 lists the domain and summary scores in the WHODAS 2.0 according to the injury type. Except in domain 2 (mobility), the summary scores were significantly higher for the participants with TBI than those with SCI, indicating that the former experience greater disability in life activities and participation than the latter do. That is, patients with TBI experience greater difficulty in most domains of daily life, such as cognition, self-care, relationships, life activities, and participation in society. WHODAS 2.0 scores exhibited a ceiling effect, such as in domain 5 (life activities), particularly in patients with severe TBI.

**Table 2:** Comparison on the basis of World Health Organization Disability Assessment Schedule (WHODAS) 2.0 scores of overall disability across domains among participants with TBI and SCI.

Variables	TBI		SCI		*p*-value
	Mean	SD	Mean	SD	
Cognition	68.84	33.244	30.69	31.891	<0.001 *
Mobility	67.48	33.591	67.50	30.013	0.986
Self-care	47.55	39.361	39.77	35.921	<0.001 *
Relationships	72.53	33.586	45.95	34.794	<0.001 *
Life activities	79.27	34.125	74.04	36.581	<0.001 *
**Participation**	62.99	27.655	58.38	26.199	<0.001 *
WHODAS 2.0 summary score	66.38	26.953	52.00	23.902	<0.001 *

* *p* < 0.05, d11–d68 (exclude d5-2 (d55–d58)) scoring by transformed WHODAS score divided by the maximum of d11–d68 score times 100.

### 3.3. WHODAS 2.0 scores and associated factors

The independent factors influencing WHODAS 2.0 scores were age, sex, injury type, socioeconomic status, place of residence, and severity of disability. All factors were adjusted in the Poisson regression model. The participants with TBI were significantly more likely to have a higher WHODAS 2.0 summary score than those with SCI (OR = 1.178, *p* < 0.05). The participants with TBI experienced significantly (*p* < 0.05) more difficulty in domain 1, cognition (OR = 1.988); domain 3, self-care (OR = 1.073); domain 4, relationships (OR = 1.429); domain 5, life activities (OR = 1.029); and domain 6, participation in society (OR = 1.016) than did participants with SCI. However, the participants with TBI were less likely to face difficulty in mobility than those with SCI (OR = 0.926, *p* < 0.05). Residing in rural institutions and severity of impairment were also major predictors of disability in this dataset (Table 3).

**Table 3:** Poisson regression of WHODAS 2.0 scores for participants with TBI and SCI: degree of disability and basic characteristics.

	Cognition	Mobility	Self-Care	Relationships	Life Activities	Participation	WHODAS 2.0 Summary Score
Intercept	23.399 ^*^	30.934 ^*^	17.357 ^*^	30.325 ^*^	47.105 ^*^	43.926 ^*^	32.544 ^*^
Age	1.009 ^*^	1.005 ^*^	1.008 ^*^	1.007 ^*^	1.004 ^*^	1.001 ^*^	1.005 ^*^
Sex							
Male							
Female	1.029 ^*^	1.015 ^*^	0.971 ^*^	0.97 ^*^	0.985 ^*^	1.003	1.001
Injury							
SCI							
TBI	1.988 ^*^	0.926 ^*^	1.073 ^*^	1.429 ^*^	1.029 ^*^	1.016 ^*^	1.178 ^*^
Socioeconomic status							
Average							
Middle-low & low	1.03 ^*^	1.074 ^*^	1.095 ^*^	0.996	1.076 ^*^	1.037 ^*^	1.047 ^*^
Urbanization level							
Urban (reference)							
Suburban	0.999	1	1.096 ^*^	0.972 ^*^	1.002	1.01	1.006
Rural	1.033 ^*^	1.028 ^*^	1.049 ^*^	1.006	1.015 ^*^	1.036 ^*^	1.028 ^*^
Place of Residence							
Community dwelling							
Institutional dwelling	1.249 ^*^	1.254 ^*^	1.427 ^*^	1.195 ^*^	1.187 ^*^	1.243 ^*^	1.247 ^*^
Severity of impairment							
Mild (reference)							
Moderate	1.368 ^*^	1.325 ^*^	1.5 ^*^	1.362 ^*^	1.206 ^*^	1.227 ^*^	1.3 ^*^
Severe	1.539 ^*^	1.601 ^*^	1.648 ^*^	1.521 ^*^	1.337 ^*^	1.297 ^*^	1.453 ^*^
Extreme	1.899 ^*^	1.723 ^*^	1.86 ^*^	1.816 ^*^	1.36 ^*^	1.414 ^*^	1.631 ^*^

^*^ *p*-value < 0.05.

## 4. Discussion

To the best of our knowledge, this is the first study evaluating the disability properties of the WHODAS 2.0 in patients with TBI and those with SCI. This study provides data that enhance our knowledge of the different function and disability between TBI and SCI patients by using the WHODAS 2.0. The study referenced a registry established by the Ministry of Health and Welfare in Taiwan for disability evaluation and functional assessment. The national health registry includes data on sociodemographic and disability-related details and the functional score in the WHODAS 2.0 [17]. The six WHODAS 2.0 domains provide crucial information that can distinguish patients with TBI and those with SCI in the components of life activities and participation. Patients with TBI have poorer cognition, self-care, relationships, and participation in society than those with SCI. TBI can coexist with posttraumatic stress disorder and musculoskeletal injuries, which can potentially exacerbate the early psychosocial outcome of TBI [21]. Our data show that patients with TBI are at risk of disability, such as independence in personal care, mobility, and activities of daily living. Reduced participation in society in such patients can be considered a result of the injury.

According to Chung *et al.*, patients with TBI face difficulties in lifestyle choices and relationships and have impaired cognition [12]. Patients with TBI experience years of disability, which commonly affects both mental and physical functions. Because of their mobility handicap, they must wait to be visited or are confined to their institutions [22]. Disabilities arising from TBI and SCI are multidimensional. This current study reported institutional patients received higher WHODAS 2.0 summary scores in all domains than community-dwelling patients did. Patients with TBI had higher WHODAS 2.0 summary scores than those with SCI did. Thus, patients with TBI were more likely to reside in institutions than those with SCI. Patients with TBI have long-term or lifelong disability, physical and psychological consequences as well as related healthcare [23]. Gupta emphasized the need for rehabilitation programs designed to improve functional outcomes following severe TBI and suggested that patients with TBI require additional rehabilitation to improve cognition, self-care, relationships, life activities, and participation in society [24].

The findings of our examination of outcome measures in SCI are in agreement with those of Wolf *et al.* [13] and Zee *et al.* [25]. The greatest limitations were found in the domains of mobility and life activities, and the least limitation in cognition. The patients with SCI received higher scores in mobility than those with TBI did. Patients with SCI face greater difficulties in covering long distances because of the higher energy cost that wheeling entails. Because of the resulting poor health and disability, SCI can be burdensome to patients and their families [26]. The requirements post-SCI are rehabilitation, a barrier-free environment, disabled-friendly public transportation services, and familial and financial support.

In this study, we found that the severity of impairment is an independent variable of domains 1–6 and WHODAS 2.0 summary scores in a dose-response relationship. That is, the higher the severity is, the higher the score. In the past, the majority of studies have focused on the severity, mortality, and morbidity; however, after the promulgation of the ICF framework in 2001, the focus shifted to addressing life activities and social participation. This study provides the connecting evidence between body function (b codes) and participation (d codes). Patients with a higher severity of impairment inevitably face greater difficulties in life activities and participation in society. Comprehensive rehabilitation, assistive devices, and social support are necessary to help patients with higher levels of injury.

The goal of rehabilitation is to minimize participation restrictions, enabling patients with TBI or SCI to function in society, reduce dependency, and improve life satisfaction and productivity. WHODAS 2.0 scores, which have subdivisions within each domain, reflect the comprehensive and holistic perspective on disability. The WHODAS 2.0 can be further developed and the outcome measures of participation improved to ensure that crucial factors of TBI and SCI are measured. Validation of WHODAS 2.0 for TBI and SCI is of priority in identifying WHODAS 2.0 categories in the domain of participation that are relevant to patients with TBI and SCI. The WHODAS 2.0 is a generic assessment instrument which is appropriate for assessing the complex and multifaceted disability associated with TBI and SCI; WHODAS 2.0 can be linked to WHO’s ICF as a standard framework across disease and healthcare settings. Further studies are needed to validate the WHODAS 2.0 for TBI and SCI from a multidisciplinary perspective.

## 5. Limitations

The limitations of this study are as follows. First, our study is a cross-sectional study and not a cohort study; thus, we could not explore causal effects. Second, there were numerous evaluators conducting the evaluation of WHODAS2.0 scores; nevertheless, on-job training was provided to all of the evaluators and helped them maintain good internal consistency in each domain. The inter-rater reliability and concurrent validity of the WHODAS 2.0 scale on TBI and SCI population require further study. Third, this population-based study is limited to Taiwan. Differences in cultures and educational standards among countries may cause differences in injury-related functional disability status.

## 6. Conclusions

Our study indicates that compared with those with SCI patients with TBI exhibited poor cognition, self-care, relationships, life activities, and participation in society. We found the independent factors influencing WHODAS 2.0 scores were age, sex, injury type, socioeconomic status, place of residence, and severity of impairment. WHODAS 2.0 scores reflect a comprehensive and holistic perspective on disability. Further studies are however needed to validate the WHODAS 2.0 for TBI and SCI from a multidisciplinary perspective.
